# Supplementary material for: Termination of the unfolded protein response is guided by ER stress-induced HAC1 mRNA nuclear retention
Source: Nat Commun. 2022 Oct 25;13:6331. doi: 10.1038/s41467-022-34133-8 (PMC9596429; doi:10.1038/s41467-022-34133-8)
Supplement: Supplementary file 1 — Supplementary Information [file 41467_2022_34133_MOESM1_ESM.pdf]

a

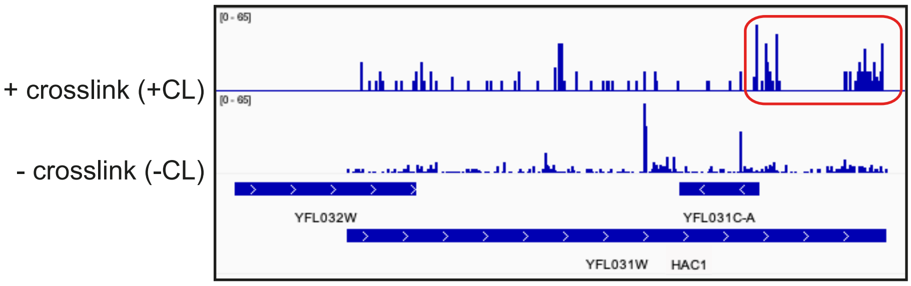

b

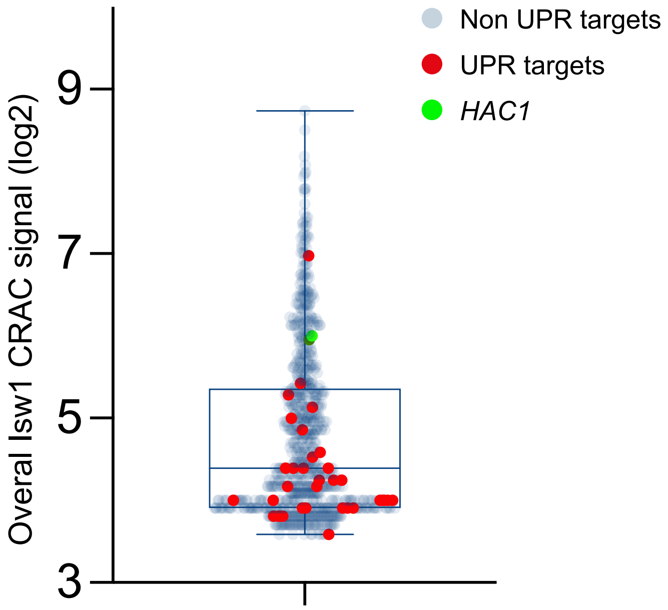

Supplementary Figure 1

a Snapshot showing the distribution of the sites of crosslink of Isw1 to the *HAC1* mRNA. These are inferred from the occurrence of one or two nucleotides deletions that are generated when the reverse transcriptase transcribes through the nucleotides covalently bound to the short peptide remaining after proteolytic digestion of the complex [25,57]. The region containing major Isw1 binding sites is boxed in red.

b Whisker plots showing the overall CRAC signal (log2) contained in the specific peaks of Isw1 binding to its RNA targets. Peaks of Isw1 binding were identified using extractPeaks[64] and only the peaks specifically present in the crosslinked sample were considered for higher specificity. Boxes extend from the 25th to 75th percentiles. The line in the middle of the box is plotted at the median. Whiskers are plotted down to the minimum and up to the maximum value, and each individual value is plotted as a point superimposed on the graph, corresponding to a transcript. All yeast genes have been included in the analysis. UPR targets and *HAC1* are shown in red and green respectively. Two independent experiments have been performed.

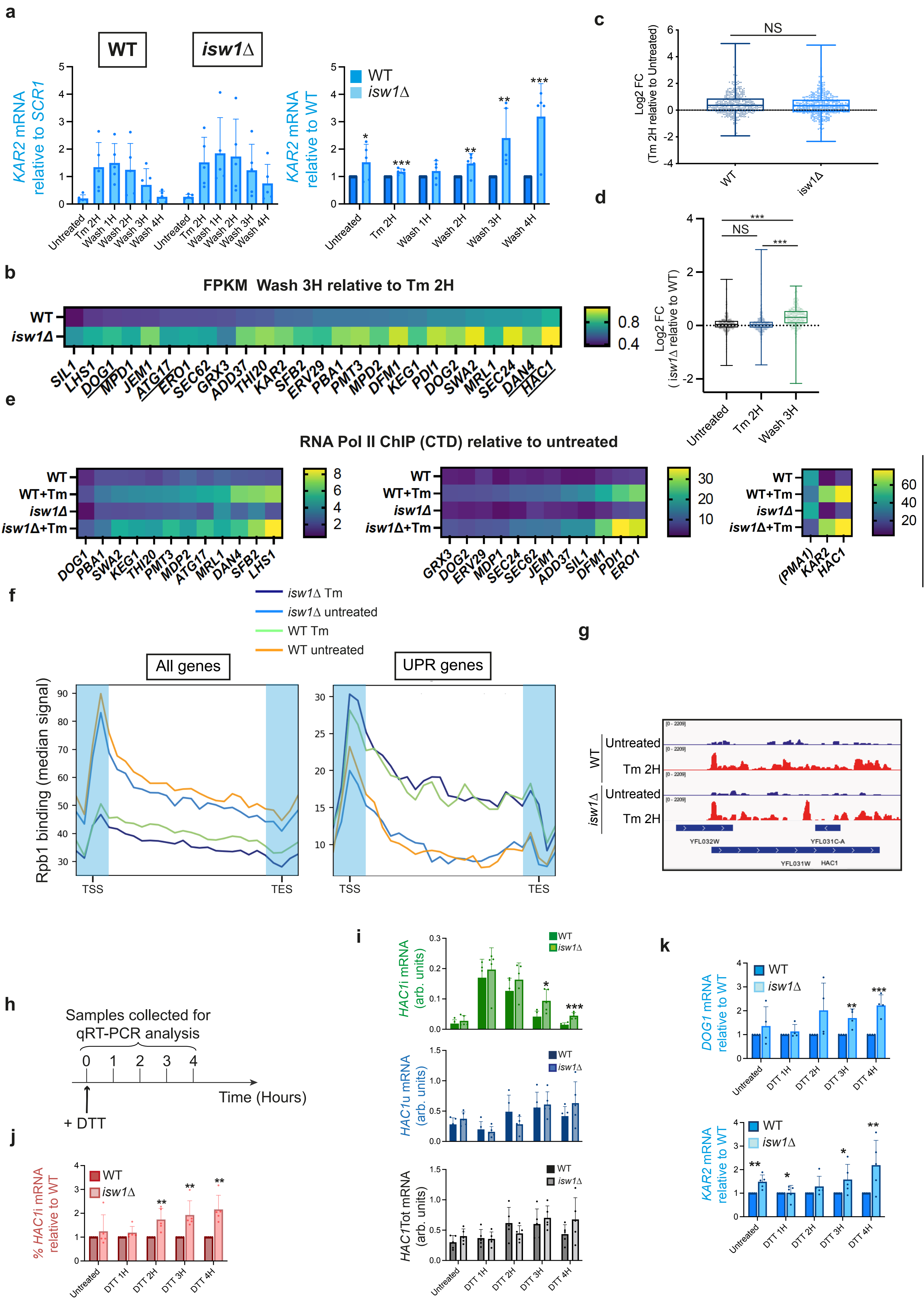
a
WT
isw1Δ
KAR2 mRNA relative to SCR1
Untreated
Tm 2H
Wash 1H
Wash 2H
Wash 3H
Wash 4H
KAR2 mRNA relative to WT
WT
isw1Δ
c
Log2 FC (Tm 2H relative to Untreated)
NS
WT
isw1Δ
d
Log2 FC (isw1Δ relative to WT)
NS
***
Untreated
Tm 2H
Wash 3H
b
FPKM Wash 3H relative to Tm 2H
WT
isw1Δ
0.8
0.4
SIL1 LHS1 DOG1 MPD1 JEM1 ATG17 ERO1 SEC62 GRX3 ADD37 THI20 KAR2 SFB2 ERV29 PBA1 PMT3 MPD2 DFM1 KEG1 PDI1 DOG2 SWA2 MRL1 SEC24 DAN4 HAC1
e
RNA Pol II ChIP (CTD) relative to untreated
WT
WT+Tm
isw1Δ
isw1Δ+Tm
DOG1 PBA1 SWA2 KEG1 THI20 PMT3 MDP2 ATG17 MRL1 DAN4 SFB2 LHS1
GRX3 DOG2 ERV29 MDP1 SEC24 SEC62 JEM1 ADD37 SIL1 DFM1 PDI1 ERO1
(PMA1) KAR2 HAC1
f
isw1Δ Tm
isw1Δ untreated
WT Tm
WT untreated
All genes
UPR genes
Rpb1 binding (median signal)
TSS
TES
g
WT
isw1Δ
Untreated
Tm 2H
YFL032W
YFL031C-A
YFL031W HAC1
h
Samples collected for qRT-PCR analysis
0 1 2 3 4
+ DTT
Time (Hours)
i
HAC1i mRNA (arb. units)
HAC1u mRNA (arb. units)
HAC1Tot mRNA (arb. units)
WT
isw1Δ
Untreated
DTT 1H
DTT 2H
DTT 3H
DTT 4H
j
% HAC1i mRNA relative to WT
k
DOG1 mRNA relative to WT
KAR2 mRNA relative to WT

Supplementary Figure 2

a The mRNA expression level of Hac1 targets is prolonged in *isw1Δ* cells compared to WT during Tm UPR time course. qRT-PCR analysis of *KAR2* expression level in *isw1Δ* cells relative to WT during Tm UPR time course. n = 5 independent experiments, mean ± sd. Unpaired one-tailed t-tests (p values relative to WT: 5,48E-03, 7,09E-03, 2,94E-02, 2,67E-03, 2,41E-02 and 2,05E-03 for Untreated, Tm 2H, Wash 1H, Wash 2H, Wash 3H and Wash 4H respectively).
b Heat maps comparing the expression fold change of 25 of the most robustly induced UPR targets 3h after drug removal relative to 2 H Tm treatment in WT and *isw1Δ* cells. For each cell type, the ratio between average normalized FPKM of three biological replicates at Tm 2 H and Wash 3 H is plotted. All genes were differentially expressed at Tm 2 H. Bold and underline indicate genes that were still differentially expressed upon Tm wash in *isw1Δ* and WT cells respectively.
c Whisker plots comparing the expression fold change of all UPR targets (defined in 11) after 2 H Tm treatment relative to untreated conditions in WT and *isw1Δ* cells. Boxes extend from the 25th to 75th percentiles. The line in the middle of the box is plotted at the median. Whiskers are plotted down to the minimum and up to the maximum value, and each individual value is plotted as a point superimposed on the graph. n= 3 independent biological replicates. Unpaired two-tailed t-test (p value *isw1Δ* relative to WT: 0,2700).
d Whisker plots comparing the expression fold change of all UPR targets between WT and *isw1Δ* cells in untreated, Tm 2 H and Wash 3 H conditions.Boxes extend from the 25th to 75th percentiles. The line in the middle of the box is plotted at the median. Whiskers are plotted down to the minimum and up to the maximum value, and each individual value is plotted as a point superimposed on the graph. n= 3 independent biological replicates. Unpaired two-tailed t-tests (p values relative to Untreated: 0,9806 and < 0,0001 for Tm 2H and Wash 3H rsepectively. (p value Wash 3H relative to Tm 2H : <0,0001).
e Heat maps showing RNA Polymerase II occupancy as determined by ChIP on the 25 most robustly induced UPR target genes depicted in Fig S2b and *PMA1*, in WT and *isw1Δ* cells. Values (mean, n=3 independent experiments), expressed as a percentage of IP and normalized to an intergenic region (HO). Three heat maps, separated according to the Pol II recruitment values upon Tm (1 µg/mL, 2 hours), are shown to facilitate reading . *PMA1* is a non UPR target. All values are available in the source data file.
f Metagene analysis of median RNAPII CRAC signal to all yeast genes or to UPR genes in WT and *isw1Δ* cells in the absence of stress or in the presence of a 2 hours Tm treatment. Features were scaled with the exception of 200 nucleotides regions centered around the transcription start site (TSS) and the transcription end site (TES) (blue-shaded areas).
g Read coverage (total hit densities per million mapped reads) determined by CRAC, illustrating the distribution of RNAPII on the *HAC1* locus in WT and *isw1Δ* cells grown under normal conditions (blue) or after a two-hours Tm treatment (red).
h DTT time course experimental setting: WT and *isw1Δ* exponentially growing cells were treated with 2 mM DTT and samples were collected for analysis at t = 0 (Untreated), 1 H, 2 H, 3 H and 4 H.
i qRT-PCR analysis of the expression levels of *HAC1*i, *HAC1*u and *HAC1*Tot in WT and *isw1Δ* cells during the DTT UPR time course depicted in d. n = 4 independent experiments, mean ± sd. Unpaired one-tailed t-tests (*p* values relative to WT: 1,81E-01, 2,71E-01, 1,32E-01, 1,14E-02, 2,37E-04 for *HAC1*i, 1,11E-01, 2,89E-018,13E-02, 3,71E-01,1,24E-01 for *HAC1*u and 9,81E-02 4,38E-01, 1,12E-01,2,46E-01,1,00E-01 for *HAC1*Tot).
j *HAC1* splicing is sustained in *isw1Δ* compared to WT cells during DTT UPR time course. The percentage of spliced *HAC1* (*HAC1*i / *HAC1*Tot x100) was calculated from the values obtained in i. n=4 independent experiments, mean ± sd. Unpaired one-tailed t-tests (p values relative to WT: 2,19E-01, 6,74E-02, 3,71E-03, 4,03E-031 and 11E-03 for Untreated, DTT 1H, DTT 2H, DTT 3H, DTT 4H respectively).
k The mRNA expression level of Hac1 transcriptional targets is prolonged in isw1Δ cells compared to WT during DTT UPR time course. qRT-PCR analysis of *DOG1* and *KAR2* expression level in *isw1Δ* cells relative to WT during DTT UPR time course. n=4, mean ± sd. Unpaired one-tailed t-tests (p values relative to WT for *DOG1*: 2,02E-01, 1,95E-01, 6,25E-02, 5,51E-03 and 7,33E-04 for Untreated, DTT 1H, DTT 2H, DTT 3H, DTT 4H respectively. p values relative to WT for *KAR2*: 2,75E-03, 4,41E-01, 8,52E-02, 3,89E-02 and 1,76E-02 for Untreated, DTT 1H, DTT 2H, DTT 3H, DTT 4H respectively).

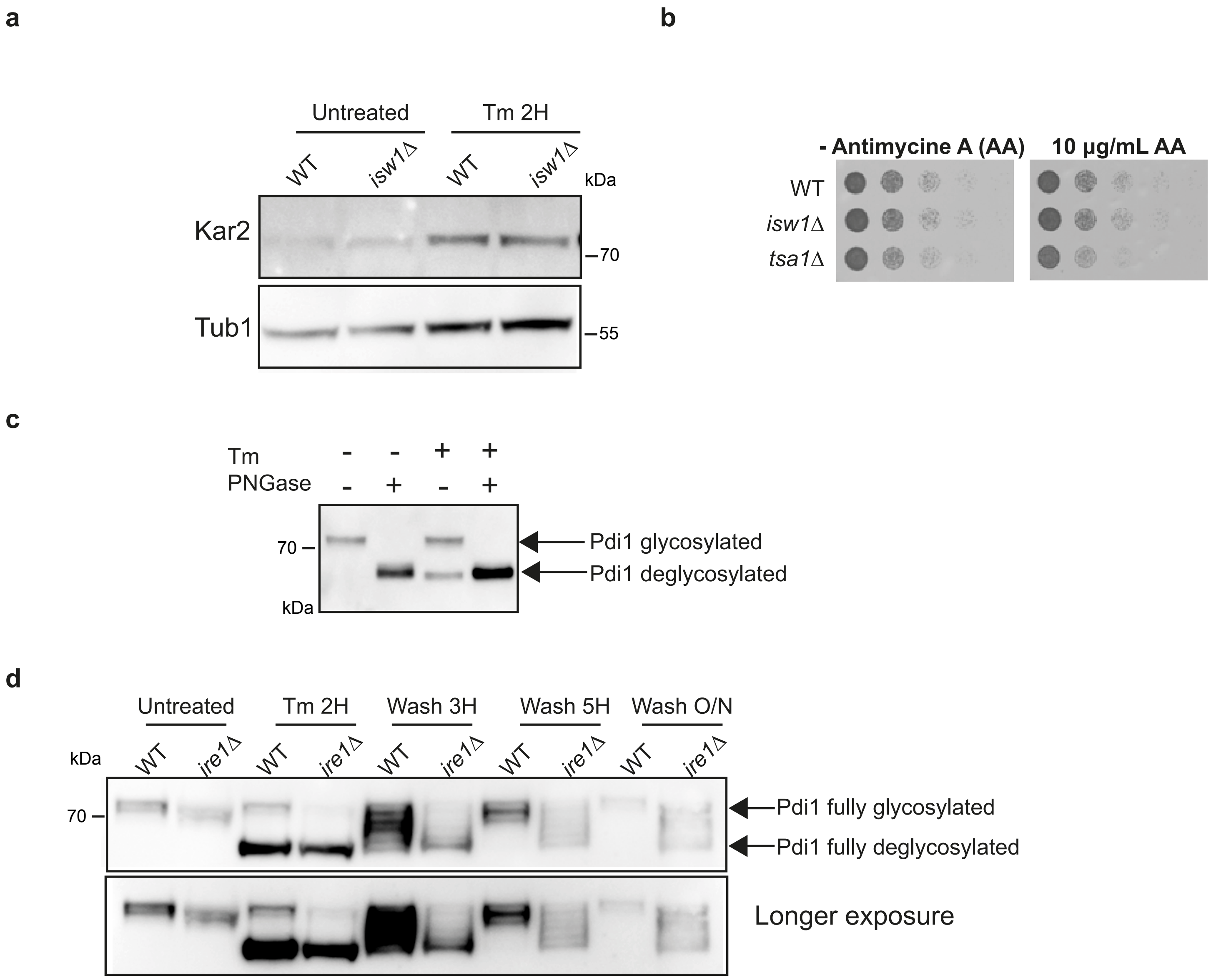

Supplementary Figure 3

a Kar2 is similarly induced by Tm in WT and *isw1*Δ cells. Total protein extracts from WT and *isw1*Δ cells treated or not with 1 µg/mL Tm for 2 H were analyzed by western blot with anti-Kar2 and Tub1 (loading) antibodies. Representative of three independent experiments.

b Fivefold serial dilutions of the indicated strains grown for 2 days at 30°C with or without 10 µg/mL of the inhibitor of oxidative phosphorylation AntimycineA (AA). *tsa1*Δ mutant serves as positive controls for the effect of the drug. Three independent experiments were performed with similar results.

c Pdi1 is a glycosylated protein. Total protein extracts from wild-type cells treated (+) or not (-) with 1 µ g/mL Tm for 1 H were resolved by reducing SDS PAGE prior to (+) or not (-) deglycosylation with PNGase and immunoblotted against Pdi1. The blot is representative of three independent experiments.

d Pdi1 re-glycosylation is a proxy for ER homeostasis reestablishment. Total protein extracts from WT and *ire1*Δ cells were analyzed by western blot for Pdi1 glycosylation during Tm UPR time course. Three independent experiments were performed with similar results.

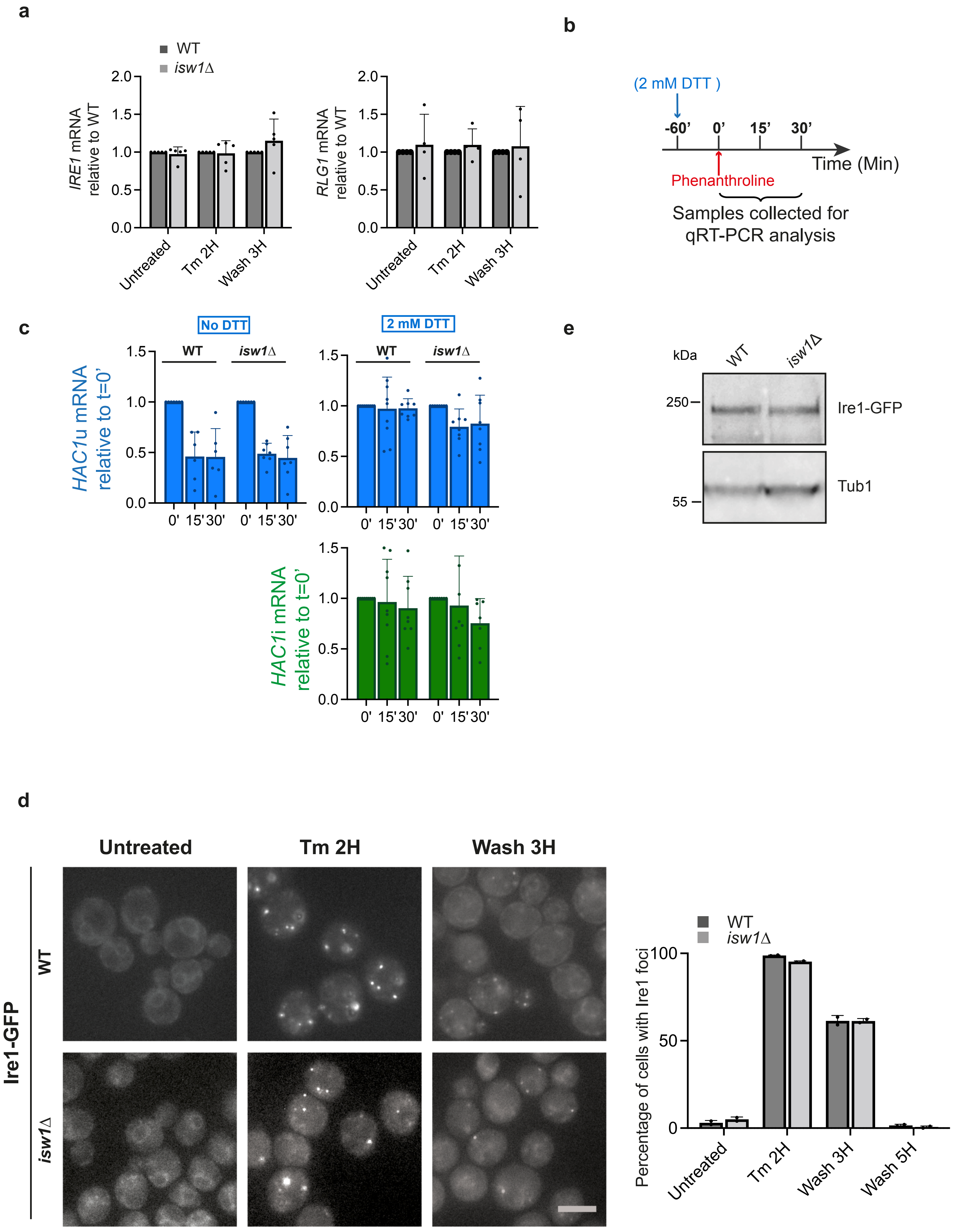

a
WT
isw1Δ
IRE1 mRNA relative to WT
RLG1 mRNA relative to WT
Untreated
Tm 2H
Wash 3H
b
(2 mM DTT)
-60'
0'
15'
30'
Time (Min)
Phenanthroline
Samples collected for qRT-PCR analysis
c
No DTT
2 mM DTT
WT
isw1Δ
HAC1u mRNA relative to t=0'
HAC1i mRNA relative to t=0'
0' 15' 30'
e
kDa
250
55
Ire1-GFP
Tub1
d
Untreated
Tm 2H
Wash 3H
Ire1-GFP
WT
isw1Δ
Percentage of cells with Ire1 foci
Wash 5H

Supplementary Figure 4 Expression of *HAC1* splicing machinery, stability of *HAC1* mRNA and kinetics of Ire1 cluster assembly and disassembly are unaffected by *ISW1* inactivation

a Inactivation of *ISW1* does not affect the steady-state level of *IRE1* and *RLG1* transcripts. The expression level of *IRE1* and RLG1 during Tm UPR TC was measured by qRT-PCR in the indicated strains. n = 4 independent experiments, mean ± sd. Unpaired one-tailed t-test (*p* values relative to WT: 2,85E-01, 4,18E-01, 1,37E-01 for *IRE1* and 3,22E-01, 2,02E-01, 3,89E-01 for *RLG1* in Untreated,Tm 2H and Wash 3H conditions.

b Analysis of transcripts stability. *isw1*Δ and WT cells were treated or not with DTT for 1 H and transcription was inhibited by addition of 0,2 mg/mL phenanthroline (t=0). Samples were collected for analysis at t = 0', 15' and 30'.

c Inactivation of *ISW1* does not affect the stability of the *HAC1* transcript in the presence or absence of DTT. WT and *isw1*Δ cells show similar *HAC1*u and *HAC1*i mRNA levels normalized to *SCR1* as analyzed by qRT-PCR. n=6 independent experiments, mean ± sd. Unpaired one-tailed t-test (*p* values relative to WT: 3,95E-01, 4,72E-01 at 15' and 30' for *HAC1*u No DTT, 8,87E-02, 7,96E-02 for *HAC1*u +DTT and 4,38E-01, 4,17E-01 for *HAC1*i +DTT.

d The kinetics of Ire1 clusterization/ declusterization is not affected by *ISW1* inactivation. Ire1-GFP localization was monitored during a Tm UPR time course and the percentage of cells with Ire1 foci was quantified in at least 400 cells per experiment. n = 2 independent experiments with similar results, mean. Scale bar, 5 µm.

e Similar expression of Ire1 in WT and *isw1*Δ cells. Total protein extracts from WT and *isw1*Δ cells expressing pRS315 IRE1-GFP were analyzed by western blot with anti-GFP or anti-tubulin (loading) antibodies. Three independent experiments were performed with similar results.

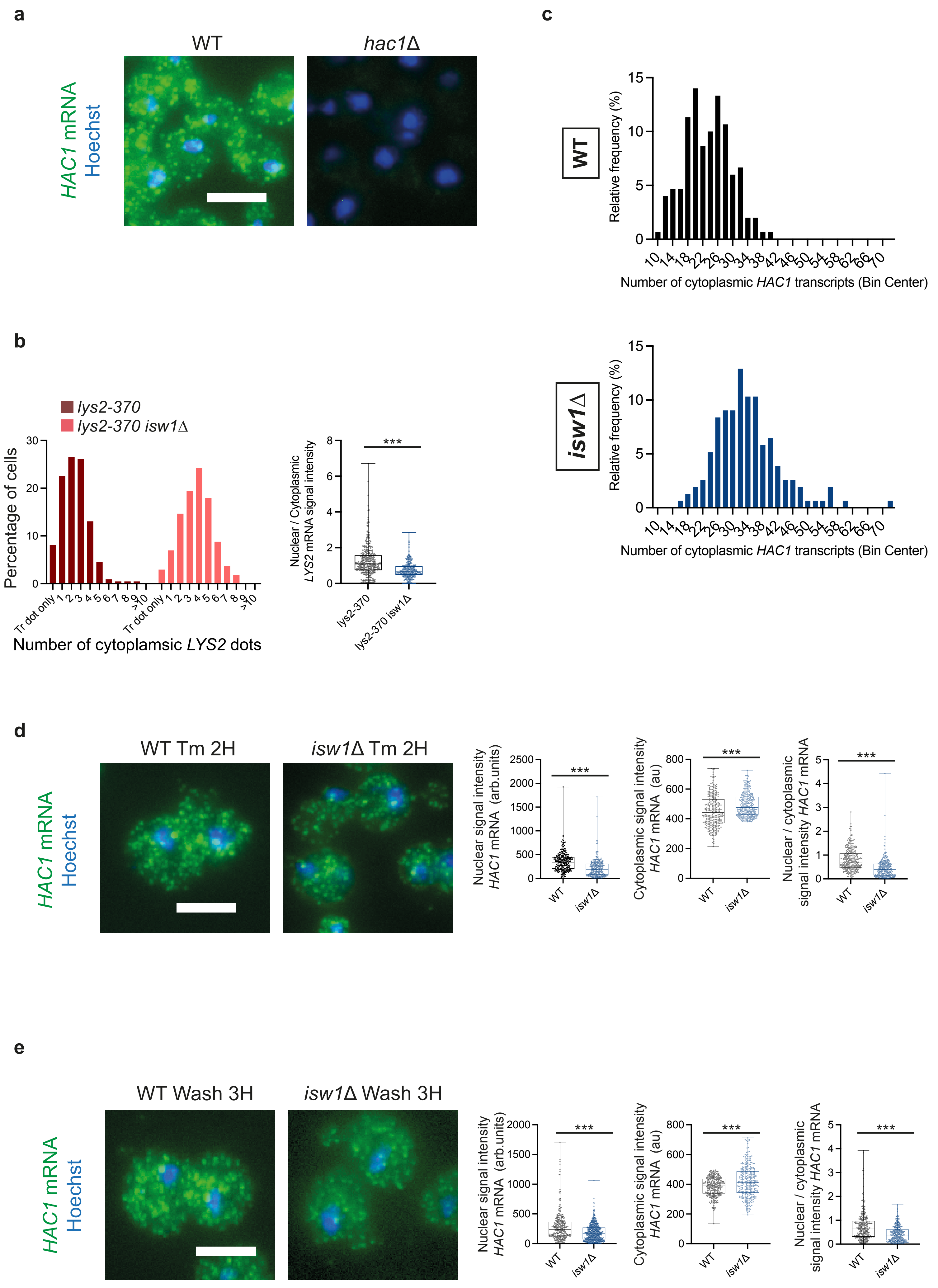

a
WT
hac1Δ
HAC1 mRNA
Hoechst
b
lys2-370
lys2-370 isw1Δ
Percentage of cells
Tr dot only
>10
Number of cytoplamsic LYS2 dots
Nuclear / Cytoplasmic LYS2 mRNA signal intensity
***
lys2-370
lys2-370 isw1Δ
c
WT
Relative frequency (%)
Number of cytoplasmic HAC1 transcripts (Bin Center)
isw1Δ
Relative frequency (%)
Number of cytoplasmic HAC1 transcripts (Bin Center)
d
WT Tm 2H
isw1Δ Tm 2H
HAC1 mRNA
Hoechst
Nuclear signal intensity HAC1 mRNA (arb.units)
Cytoplasmic signal intensity HAC1 mRNA (au)
Nuclear / cytoplasmic signal intensity HAC1 mRNA
WT
isw1Δ
***
e
WT Wash 3H
isw1Δ Wash 3H
HAC1 mRNA
Hoechst
Nuclear signal intensity HAC1 mRNA (arb.units)
Cytoplasmic signal intensity HAC1 mRNA (au)
Nuclear / cytoplasmic signal intensity HAC1 mRNA
WT
isw1Δ
***

Supplementary Figure 5

a Quasar®670-*HAC1* probes specificity. *HAC1* mRNA subcellular localization was analyzed by FISH in WT and *hac1*Δ cells. Scale bar, 5 µm.Two independent experiments were performed with similar results.

b Validation of the *HAC1* mRNA FISH quantification approach. The abundance of the *HAC1* mRNA (ranked among the top 100 most expressed mRNA) prevented the accurate identification of individual transcript and prompted us to quantify the intensities of the *HAC1* mRNA nuclear and cytoplasmic signals, as a proxy for absolute nuclear and cytoplasmic *HAC1* mRNA quantification. This approach was applied to a previously published[26] and manually counted data set, in which the number of cytoplasmic *LYS2* transcripts, a weakly expressed Isw1 target, was evaluated in a mutant defective for *LYS2* mRNA nuclear export (*lys2-370*) inactivated (*lys2-370 isw1*Δ) or not for *ISW1*. The decrease in the percentage of cells with only nuclear localized *LYS2* at the expense of an increase in the percentage of cells with 3 or more cytoplasmic dots observed upon *ISW1* inactivation is mirrored by a significant decrease in the nuclear to cytoplasmic *LYS2* mRNA signal ratio. 300 independent cells were evaluated. Unpaired two-tailed t-test (*p* value<0,0001). Nuclear to cytoplasmic signal intensities were plotted into whisker plots. Boxes extend from the 25th to 75th percentiles. The line in the middle of the box is plotted at the median. Whiskers are plotted down to the minimum and up to the maximum value, and each individual value is plotted as a point superimposed on the graph. Two independent experiments were performed.

c The number of cytoplasmic *HAC1* transcripts was evaluated by manual counting of about 150 cells per strain. Note that all cells displayed a unique nuclear dot of variable intensity, corresponding to the transcription site, at which various amounts of nuclear-retained RNAs congregate. This method is therefore complementary to the global intensity quantification approach applied in Fig. 4c. The frequency distribution of cytoplasmic *HAC1* transcripts per bin (width 2) is represented for WT and *isw1*Δ. Two independent experiments were evaluated with similar results.

d, e *HAC1* mRNA subcellular localization was analyzed and quantified as in Fig. 4c in WT and *isw1*Δ cells after 2 H Tm treatment and 3 H after drug removal. Nuclear and cytoplasmic Quasar®670-*HAC1* signal intensities were measured in at least 200 independent cells. Unpaired two-tailed t-test (*p* value<0,0001 for Tm 2H and Wash 3H). Signal intensities were plotted into whisker plots. Boxes extend from the 25th to 75th percentiles. The line in the middle of the box is plotted at the median. Whiskers are plotted down to the minimum and up to the maximum value, and each individual value is plotted as a point superimposed on the graph. Two independent experiments were performed. Scale bar, 5 µm.

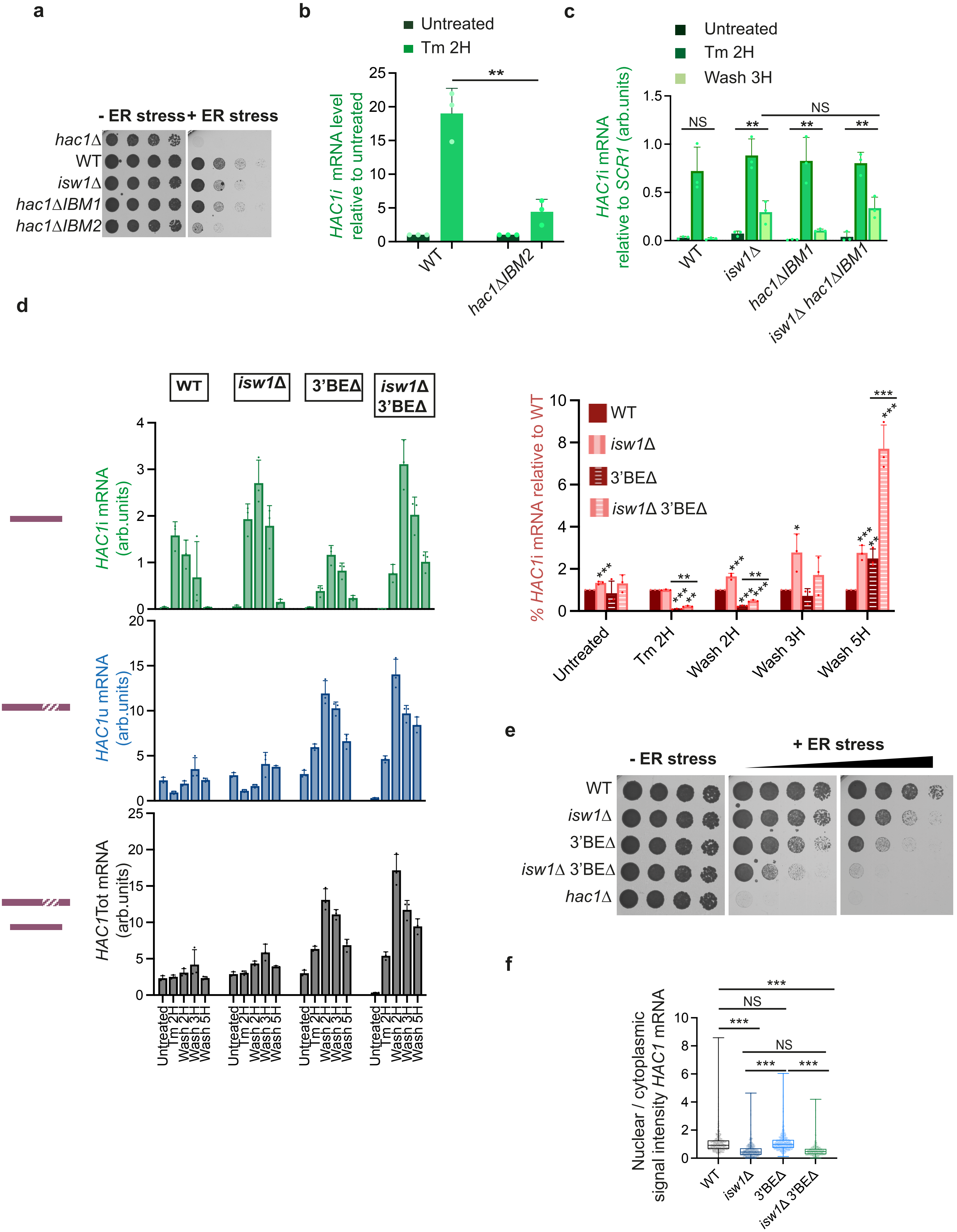
a
- ER stress
+ ER stress
hac1Δ
WT
isw1Δ
hac1ΔIBM1
hac1ΔIBM2
b
Untreated
Tm 2H
HAC1i mRNA level relative to untreated
**
WT
hac1ΔIBM2
c
Untreated
Tm 2H
Wash 3H
HAC1i mRNA relative to SCR1 (arb.units)
NS
**
WT
isw1Δ
hac1ΔIBM1
isw1Δ hac1ΔIBM1
d
WT
isw1Δ
3'BEΔ
isw1Δ 3'BEΔ
HAC1i mRNA (arb.units)
HAC1u mRNA (arb.units)
HAC1Tot mRNA (arb.units)
Untreated
Tm 2H
Wash 2H
Wash 3H
Wash 5H
% HAC1i mRNA relative to WT
WT
isw1Δ
3'BEΔ
isw1Δ 3'BEΔ
***
**
*
Untreated
Tm 2H
Wash 2H
Wash 3H
Wash 5H
e
- ER stress
+ ER stress
WT
isw1Δ
3'BEΔ
isw1Δ 3'BEΔ
hac1Δ
f
Nuclear / cytoplasmic signal intensity HAC1 mRNA
***
NS
WT
isw1Δ
3'BEΔ
isw1Δ 3'BEΔ

Supplementary Fig. 6

a *hac1*Δ IBM2 cells are hypersensitive to Tm. Fivefold serial dilutions of the indicated strains grown for 3 days at 30°C with (+ER stress) or without (- ER stress) 0,4 µg/mL Tm.

b *hac1*Δ IBM2 cells are compromised for UPR induction. *HAC1*i mRNA level normalized by *SCR1* upon 2 H Tm treatment relative to untreated conditions as evaluated by qRT-PCR analysis. n = 3 independent experiments, mean ± sd. Unpaired one-tailed t-test (*p* value *hac1*Δ IBM2 relative to WT: 1,86E-03).

c *HAC1*i relative to *SCR1* mRNA expression during Tm UPR time courses, as evaluated by qRT-PCR in the indicated strains. n = 4 independent experiments, mean ± sd. Unpaired one-tailed t-test (*p* values relative to Untreated: 1,50E-01, 1,64E-02, 1,42E-04, 7,15E-03 for WT, *isw1*Δ, *hac1* ΔIBM1 and *isw1*Δ*hac1*ΔIBM1 respectively. *p* value *isw1*Δ*hac1*ΔIBM1 relative to *isw1*Δ: 3,41E-01).

d qRT-PCR analysis of the expression levels of *HAC1*i, *HAC1*u, and *HAC1*Tot and of the percentage of spliced *HAC1* in WT, i*sw1*Δ, *hac1*Δ 3'BE and *isw1*Δ, *hac1*Δ 3'BE cells during Tm UPR time course. n = 3 independent experiments, mean ± sd. Unpaired one-tailed t-tests (*p* values relative to WT: 4,80E-04, 3,77E-01, 7,64E-04, 1,25E-02, 5,13E-04 for *isw1*Δ, 3,34E-01, 8,06E-08, 8,33E-07 1,17E-01, 2,25E-03 for *hac1*Δ 3'BE, 1,25E-01, 4,78E-07, 1,20E-05, 1,21E-01, 2,42E-04 for *isw1*Δ, *hac1*Δ 3'BE. *p* values *isw1*Δ, *hac1*Δ 3'BE relative to *hac1*Δ 3'BE: 1,57E-01, 1,37E-03, 5,53E-04, 7,50E-02, 8,51E-04 for Untreated,Tm 2H, Wash 2H, Wash 3H, Wash 5H respectively).

e Fivefold serial dilutions of the indicated strains grown for 3 days at 30°C in the presence (+ ER stress) or not (- ER stress) of 0,4 µg/mL or 0,6 µg/mL Tm.

f *HAC1* mRNA localization was analyzed by FISH using Quasar®670-HAC1 probes in the indicated strains and quantified as in Fig. 4c. Nuclear and cytoplasmic Quasar®670-*HAC1* signal intensities were measured in at least 200 independent cells. Nuclear to cytoplasmic ratio were plotted into min to max whiskers plots. Boxes extend from the 25th to 75th percentiles. The line in the middle of the box is plotted at the median. Whiskers are plotted down to the minimum and up to the maximum value, and each individual value is plotted as a point superimposed on the graph. Two independent experiments were performed with similar results. Unpaired two-tailed t-tests (*p* values relative to WT: <0,0001, 0,1063 and <0,0001 for *isw1*Δ, *hac1*Δ 3'BE and *isw1*Δ *hac1*Δ 3'BE respectively. *p* value *isw1*Δ *hac1*Δ 3'BE relative to *isw1*Δ: 0,8035. *p* values relative to *hac1*Δ 3'BE: <0.0001 for *isw1*Δ and *isw1*Δ *hac1*Δ 3'BE.) Two independent experiments were performed with similar results.

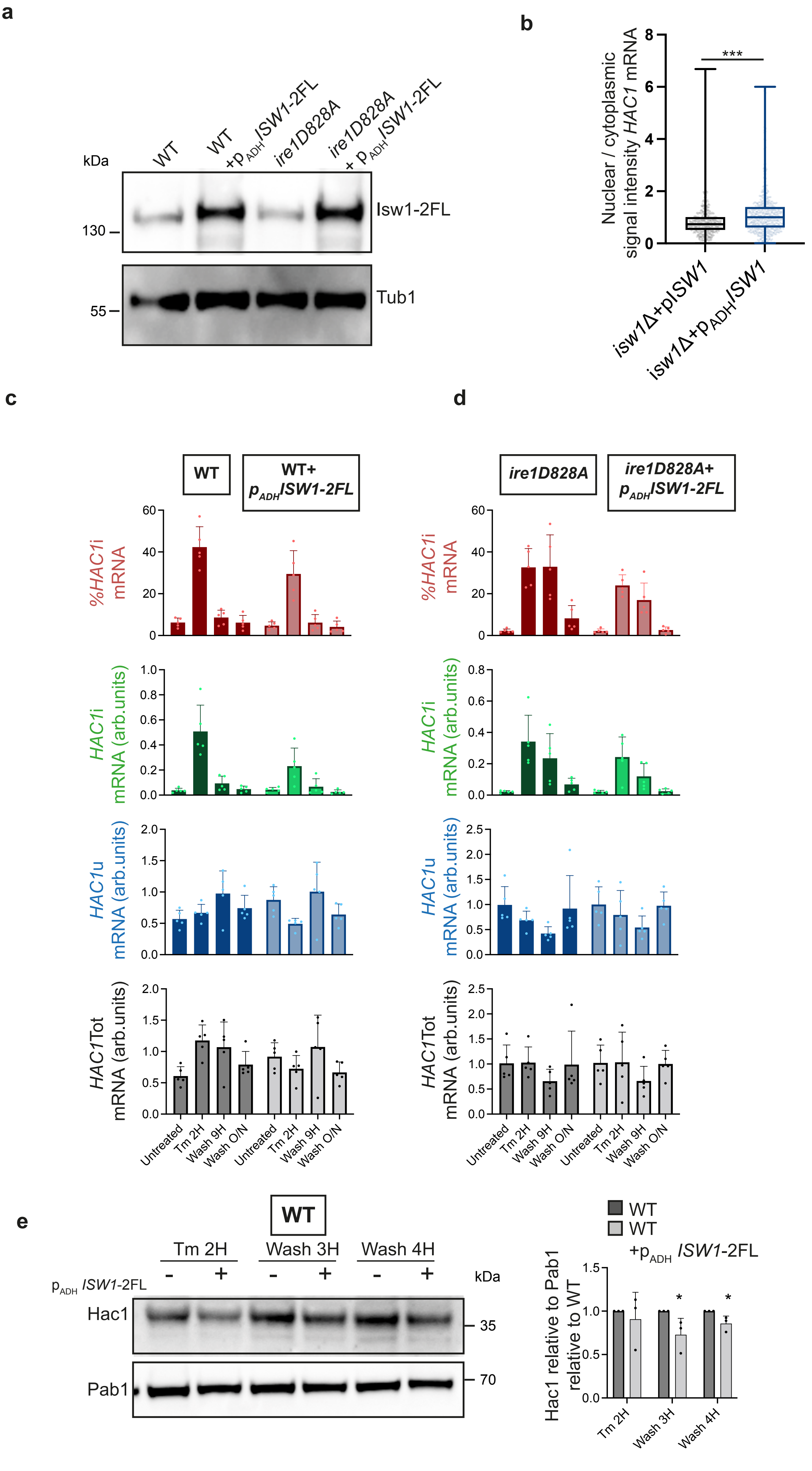
a
kDa
WT
WT +pADH ISW1-2FL
ire1D828A
ire1D828A + pADH ISW1-2FL
130
55
Isw1-2FL
Tub1
b
Nuclear / cytoplasmic signal intensity HAC1 mRNA
***
isw1Δ+pISW1
isw1Δ+pADH ISW1
c
d
WT
WT+ pADH ISW1-2FL
ire1D828A
ire1D828A+ pADH ISW1-2FL
%HAC1i mRNA
HAC1i mRNA (arb.units)
HAC1u mRNA (arb.units)
HAC1Tot mRNA (arb.units)
Untreated
Tm 2H
Wash 9H
Wash O/N
e
WT
Tm 2H
Wash 3H
Wash 4H
pADH ISW1-2FL
Hac1
Pab1
kDa
35
70
WT
WT +pADH ISW1-2FL
Hac1 relative to Pab1 relative to WT
*

Supplementary Figure 7

a Isw1 overexpression. Total protein extracts from WT and *ire1D828A* cells overexpressing or not Isw1-2FL were analyzed by western blot with anti-FLAG anti-Tubulin (loading). 3 independent experiments were performed with similar results.

b The effect of Isw1 overexpression on *HAC1* mRNA subcellular localization was analyzed by RNA FISH using Quasar®670-*HAC1* probes in the indicated strains and quantified as in Fig. 4c.Nuclear and cytoplasmic Quasar®670-*HAC1* signal intensities were measured in at least 200 independent cells. Unpaired two-tailed t-test (*p* value<0,0001). Nuclear to cytoplasmic intensities ratio were plotted into min to max whiskers plots. Boxes extend from the 25th to 75th percentiles. The line in the middle of the box is plotted at the median. Whiskers are plotted down to the minimum and up to the maximum value, and each individual value is plotted as a point superimposed on the graph.Two independent experiments were performed.

c, d Isw1 overexpression impairs *HAC1* mRNA splicing. qRT-PCR analysis of the expression levels of *HAC1*i, *HAC1*u, and *HAC1*Tot normalized to *SCR1* in WT and *ire1D828A* cells overexpressing or not *ISW1* during Tm UPR time courses. n = 5 independent experiments, mean ± sd.

e *ISW1* overexpression limits Hac1 expression. Total protein extracts, prepared from WT cells, overexpressing *ISW1* (+p_{ADH}*ISW1*) or not, during Tm time-courses were analyzed by western blot with anti-Hac1 and Pab1 (loading) antibodies. The level of Hac1 and Pab1 was quantified. n = 3 independent experiments, mean ± sd. Unpaired one-tailed t-tests (*p* values: 3,15E-01, 3,34E-02, 2,27E-02 for Tm 2H, Wash 2H, Wash 4H respectively)

a

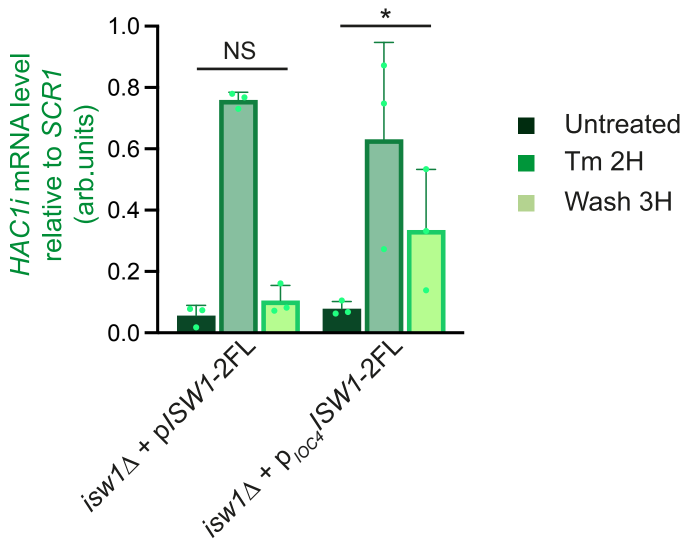

Supplementary Fig. 8

a Cells expressing non-UPR-inducible *ISW1* (p_{IOC4}*ISW1*) are defective for UPR termination. *HAC1*i normalized to *SCR1* mRNA expression during Tm UPR time courses, as evaluated by qRT-PCR. n = 3, mean ± sd. Unpaired one-tailed t-tests (*p* values relative to Untreated: 1,13E-01 and 4,48E-02 for *isw1Δ* +p*ISW1*-2FL and *isw1Δ*+p_{IOC4}-2FL respectively).

Supplementary Table 1

Name	Genotype	Origin
W303	*Mat α leu2-3,112 trp1-1 can1-100 ura3-1 ade2-1 his3-11,15*	R. Haguenauer-Tsapis
hac1Δ	*Mat α leu2-3,112 trp1-1 can1-100 ura3-1 ade2-1 his3-11,15, hac1::HPH*	This study
hac1ΔIBM1	*Mat α leu2-3,112 trp1-1 can1-100 ura3-1 ade2-1 his3-11,15, hac1ΔIBM1::KanMx*	This study
hac1ΔIBM2	*Mat α leu2-3,112 trp1-1 can1-100 ura3-1 ade2-1 his3-11,15, hac1ΔIBM2::KanMx*	This study
HAC1- 3'UTR *ACT1*	*Mat α leu2-3,112 trp1-1 can1-100 ura3-1 ade2-1 his3-11,15, HAC1- 3'UTR ACT1-HA::KanMx*	This study
ire1Δ	*Mat α leu2-3,112 trp1-1 can1-100 ura3-1 ade2-1 his3-11,15, ire1::HPH*	This study
isw1Δ	*Mat α leu2-3,112 trp1-1 can1-100 ura3-1 ade2-1 his3-11,15, isw1::KanMx*	This study
hac1Δisw1Δ	*Mat α leu2-3,112 trp1-1 can1-100 ura3-1 ade2-1 his3-11,15, hac1::HPH, isw1::KanMx*	This study
isw1Δ hac1ΔIBM1	*Mat α leu2-3,112 trp1-1 can1-100 ura3-1 ade2-1 his3-11,15, isw1:: HPH, hac1ΔIBM1::KanMx*	This study
ire1Δisw1Δ	*Mat α leu2-3,112 trp1-1 can1-100 ura3-1 ade2-1 his3-11,15, ire1::HPH, isw1::KanMx*	This study

Cbp20-TAP	*Mat α leu2-3,112 trp1-1 can1-100 ura3-1 ade2-1 his3-11,15, Cbp20-TAP::TRP*	This study
Cbp20-TAP, *isw1Δ*	*Mat α leu2-3,112 trp1-1 can1-100 ura3-1 ade2-1 his3-11,15, Cbp20-TAP::TRP, isw1::KanMx*	This study
ISW1-HTP	*Mat α leu2-3,112 trp1-1 can1-100 ura3-1 ade2-1 his3-11,15, Isw1-HTP::TRP*	This study
ISW1-HTP, *HAC1*-3'UTR *ACT1*	*Mat α leu2-3,112 trp1-1 can1-100 ura3-1 ade2-1 his3-11,15, Isw1-HTP::TRP, HAC1- 3'UTR ACT1-HA::KanMx*	This study
ISW1-HTP, *ΔIBM1*	*Mat α leu2-3,112 trp1-1 can1-100 ura3-1 ade2-1 his3-11,15, Isw1-HTP::TRP,hac1ΔIBM1::KanMx*	This study
RPB1-HTP	*Mat a leu2-3,112 trp1-1 can1-100 ura3-1 ade2-1 his3-11,15, RPB1-HTP::TRP*	D. Libri
RPB1-HTP, *isw1Δ*	*Mat a leu2-3,112 trp1-1 can1-100 ura3-1 ade2-1 his3-11,15, RPB1-HTP::TRP, ISW1::KanMx*	This study
BY 4742	*MATα, his3Δ1, leu2Δ0, met15Δ0, ura3Δ0*	Euroscarf
isw1Δ	*MATα, his3Δ1, leu2Δ0, met15Δ0, ura3Δ0, isw1::KanMx*	Euroscarf
sod2Δ	*MATα, his3Δ1, leu2Δ0, met15Δ0, ura3Δ0, sod2::KanMx*	Euroscarf
hog1Δ	*MATα, his3Δ1, leu2Δ0, met15Δ0, ura3Δ0, hog1::KanMx*	Euroscarf
get1Δ	*MATα, his3Δ1, leu2Δ0, met15Δ0, ura3Δ0, get1::KanMx*	Euroscarf
tsa1Δ	*MATα, his3Δ1, leu2Δ0, met15Δ0, ura3Δ0, tsa1::KanMx*	Euroscarf

Supplementary Table 2

**Plasmid name**	**Reference or origin**
pRS416-*ISW1*-2FL	T. Tsukiyama
pRS415$_{\mathrm{ADH}}$-*ISW1*-2FL	This study
pRS416$_{\mathrm{IOC4}}$-*ISW1*-2FL	This study
pRS316-*HAC1*	M. Dey
pRS314-*IRE1*	M. Niwa
pRS314-*ire1D828A*	M. Niwa
pRS305-*IRE1*-GFP	P. Walter

Supplementary Table 3

Primer name	Target	Primer sequence
qAB120	*DOG1*	F, ATAGTGAGTACAACAGTGGCCG
qAB21	*DOG1*	R, TGCACCAGGAATAAGGCTTACT
qAB88	*HAC1*	R, CATCGTAATCACGGCTGGA
qAB91	*HAC1*	F, CCTGCCGTAGACAACAACAA
qAB135	*HAC1*	R, TCAAACCTGACTGCGCTTCT
qAB124	*HAC1*	F, TACGTTCACACCTTCACCTCTG
qAB125	*HAC1*	R, TCCGTCTTAAACATCTGCAGCT
qAB136	*IOC2*	F, ACCTCTTCAATAACCGCCCATT
qAB137	*IOC2*	R, TTCTATTGCCGTGCGAATTTCC
qAB114	*IRE1*	F, ATTTCGGGAGCCAGAGTGTG
qAB115	*IRE1*	R, TGAAGCGCTAGGGGAACATC
qAB100	*ISW1*	F, TCAGCAGATGTCGTTGTCTTGTA
qAB101	*ISW1*	R, GCTTCTTCTGACCAATACGATGC
qAB104	*KAR2*	F, TAGTTAGAGGTGCCGATGATGTAG
qAB105	*KAR2*	R, GTAAGATGGGGTGATTCTGTTACC
qAB108	*PDI1*	F, ATTGGAAGAATACAAGCCTCTCTTT
qAB109	*PDI1*	R, TTCATTAGACCTCTGTTCTTTTTGG
qAB7	*PMA1*	F, TCAGCTCATCAGCCAACTCAAG
qAB8	*PMA1*	R, CGTCGACACCGTGATTAGATTG
qAB88	*SCR1*	F, GTTCTGAAGTGTCCCGGCTATAA
qAB89	*SCR1*	R, CACCAGACAGAGAGACGGATTC
qAB140	*RLG1*	F, TGGCAAAACAACAACTTCCCAG
qAB141	*RLG1*	R, CGCGGAATTGGTGATTGTTTCT
qAB168	*SIL1*	F, TCATGGCCGCTCTGTCAAAT
qAB169	*SIL1*	R, TCGTACATGTCGGCCTTCAA
qAB170	*LHS1*	F, ACACTACTCAGCCCGTTACA
qAB171	*LHS1*	R, ACTTTGCCTTCTCTGCTGCT

qAB172	*KEG1*	F, CGTAAGGGCGTAAAGGAAAGA
qAB173	*KEG1*	R, TGCTAGTAAAACCACCCTGTTG
qAB174	*JEM1*	F, position 1546 in CAACAGCAGCAACAACACCA
qAB175	*JEM1*	R, TGTTTGTCGTTATGGTTGGCC
qAB176	GRX3	F, TAAATTGGTCAATGCCGCGC
qAB177	GRX3	R, AGTTGAGGGAAAGTTGGCCA
qAB178	*DFM1*	F, GTGCCCACAATGAAACTGCA
qAB179	*DFM1*	R, AGGGGCTGTTTGTTTGTCCT
qAB180	*DAN4*	F, AGCAACCACTTCCGCAGAAT
qAB181	*DAN4*	R, ACGGCTGAAGTGAAAGTGGA
qAB182	*ATG17*	F, AGTGCCAGTAATTGAGCGTCA
qAB183	*ATG17*	TTCAAGGTCCGTTAGTTCCCTG
qAB184	*ADD37*	F, AGGTGTGCTCGTAAAAGACCA
qAB185	*ADD37*	R, ACTGTTTTGGTGTGCTCGTT
qAB186	*SWA2*	F, AGGCGTGCTGAGTCTTTTGA
qAB187	*SWA2*	R, TTTTGTTGCAGGCGAGGTTG
qAB188	*SFB2*	F, AGCACTGGAGGCAAAGTTGA
qAB189	*SFB2*	R, AAAACGGCCCAGGTGAGATA
qAB190	*SEC24*	F, AACAATGAAGAGTCCGCCGA
qAB191	*SEC24*	R, ACCACCAACGCCACCAATTA
qAB192	*PBA1*	F, *TCTGCCCGGTTCCAAAAGTT*
qAB193	*PBA1*	R, TGGGGAAGACTTGGACTTTTCA
qAB194	*MLR1*	F, *ACGAAAACGGCTCTATGTGC*
qAB195	*MLR1*	R, TTCTCCCCGCAAATTCGACT
qAB196	*MPD2*	F, TGAAGCTGTCACGATGGTCA
qAB197	*MPD2*	R, AATCCAGGCAAGTCGGTACA
qAB198	*MPD1*	F, *ACCCAAACTTTCCGTGGTGT*
qAB199	*MPD1*	R, TATCGCTCTGTCGCTGTTCA
qAB200	*SEC62*	F, CGCGGCTCCTATTATGTGTCTC

qAB201	*SEC62*	R, TACACCGCAATCTTCGAACAGG
qAB202	*DOG2*	F, GAGCTCGTGATTTATTGCGTCA
qAB203	*DOG2*	R, CACGGAAACCTGTGTCAAATCA
qAB204	*ERV29*	F, GGGGCTCACTGACAATGCA
qAB205	*ERV29*	R, CATGGAAACGGTAACAACCACTAG
qAB206	*PMT3*	F, CTGCTTACGGAGATGTAGACTTAGG
qAB207	*PMT3*	R, CCACCAGGTTCTCTTATCCCTTTT
qAB208	*THI20*	F, TGCATCAAACTTGGCTCGTG
qAB209	*THI20*	TGGGATTTTGTCAGCAGCAC
qAB210	HO intergenic	F, GAAACCACGAAAAGTTCACCA
qAB211	HO intergenic	R, AGCTTCTGCAAACCTCATTTG

Supplementary Table 4

Antibody	Reference	Dilution
Peroxidase AffiniPure Goat Anti-Mouse IgG (H+L)	Jackson ImmunoResearch - 115-035-003	1 : 10 000
Peroxidase AffiniPure Goat Anti-Rabbit IgG (H+L)	Jackson ImmunoResearch - 115-035-144	1 : 10 000
Peroxidase AffiniPure Goat Anti-Rat IgG (H+L)	Jackson ImmunoResearch - 115-035-143	1 : 10 000
Anti FLAG-M2	SIGMA-ALDRICH - F3165	1 : 20 000
Anti-GFP	Takara - 632 381	1 : 5 000
Anti-Kar2 HDEL	Santa Cruz - sc 53472	1 : 5 000
Anti-Pdi1	Invitrogen – MAI-10032	1 : 2 000
Anti-Pab1	Abcam - ab 189635	1 : 10 000
Anti-Tub1	Santa Cruz - sc 53030	1 : 2 000
Anti-Hac1	Peter Walter [7]	1 : 8 000
Anti RNA Polymerase II (8WG16)	Eurogentec MMS-126P-050	1µl/OD
