# Supplementary material for: Termination of the unfolded protein response is guided by ER stress-induced HAC1 mRNA nuclear retention
Source: Nat Commun. 2022 Oct 25;13:6331. doi: 10.1038/s41467-022-34133-8 (PMC9596429; doi:10.1038/s41467-022-34133-8)
Supplement: Supplementary file 3 — Reporting Summary [file 41467_2022_34133_MOESM3_ESM.pdf]

nature portfolio

Corresponding author(s): Anna Babour

Last updated by author(s): Oct 8, 2022

Reporting Summary

Nature Portfolio wishes to improve the reproducibility of the work that we publish. This form provides structure for consistency and transparency in reporting. For further information on Nature Portfolio policies, see our Editorial Policies and the Editorial Policy Checklist.

Statistics

For all statistical analyses, confirm that the following items are present in the figure legend, table legend, main text, or Methods section.

n/a	Confirmed	
☐	☒	The exact sample size (*n*) for each experimental group/condition, given as a discrete number and unit of measurement
☐	☒	A statement on whether measurements were taken from distinct samples or whether the same sample was measured repeatedly
☐	☒	The statistical test(s) used AND whether they are one- or two-sided<br>*Only common tests should be described solely by name; describe more complex techniques in the Methods section.*
☒	☐	A description of all covariates tested
☒	☐	A description of any assumptions or corrections, such as tests of normality and adjustment for multiple comparisons
☐	☒	A full description of the statistical parameters including central tendency (e.g. means) or other basic estimates (e.g. regression coefficient) AND variation (e.g. standard deviation) or associated estimates of uncertainty (e.g. confidence intervals)
☐	☒	For null hypothesis testing, the test statistic (e.g. *F*, *t*, *r*) with confidence intervals, effect sizes, degrees of freedom and *P* value noted<br>*Give P values as exact values whenever suitable.*
☒	☐	For Bayesian analysis, information on the choice of priors and Markov chain Monte Carlo settings
☒	☐	For hierarchical and complex designs, identification of the appropriate level for tests and full reporting of outcomes
☒	☐	Estimates of effect sizes (e.g. Cohen's *d*, Pearson's *r*), indicating how they were calculated

Our web collection on statistics for biologists contains articles on many of the points above.

Software and code

Policy information about availability of computer code

Data collection

- Microscopy: images were aquired using a Axiovert 200M microscope (Carl Zeiss MicroImaging, Inc.) with a 63X oil immersion objective (NA =1,4) and captured using a monochrome digital camera (Axiocam MRm; Carl Zeiss MicroImaging, Inc).
- qRT-PCR was carried out on a QuantStudioTM 5, Thermo Fisher

Data analysis

- Images were analyzed with ImageJ version 2.1.0/1.53c
- Statistical analysis was performed with the Prism 9.1.2 software (GraphPad Software) or microsoft xcel 16.16.27
- RNAseq analysis was performed by Novogene as described in the method section (HISAT2 v 2.1.0, HTSeq 0.11.3, R package 3.4.2, DESeq2 1.16.1) Expression of UPR and non UPR target genes was performed using Prism 9.4.1.
- CRAC analysis was performed using: pyCRAC v1.2.2.7, Fastx reverse complement (part of the fastx toolkit, http://hannonlab.cshl.edu/fastx_toolkit/), bowtie2 (-N 1), peakCcall (Ref 64: code available upon request to DL).

For manuscripts utilizing custom algorithms or software that are central to the research but not yet described in published literature, software must be made available to editors and reviewers. We strongly encourage code deposition in a community repository (e.g. GitHub). See the Nature Portfolio guidelines for submitting code & software for further information.

Data

Policy information about availability of data

All manuscripts must include a data availability statement. This statement should provide the following information, where applicable:

- Accession codes, unique identifiers, or web links for publicly available datasets
- A description of any restrictions on data availability
- For clinical datasets or third party data, please ensure that the statement adheres to our policy

Unique biological material generated in this study is available from the corresponding author upon reasonable request.
Source data for each figure are provided with this paper as a source data file.
The SaCer3 genome was retrieved from UCSC (https://genome.ucsc.edu/).
Gene expression data have been deposited in ArrayExpress under the accession code E-MTAB-10511 [https://www.ebi.ac.uk/biostudies/arrayexpress/studies/E-MTAB-10511]. Rpb1 CRAC has been deposited to GEO under the accession code GSE207652 [https://www.ncbi.nlm.nih.gov/geo/query/acc.cgi?acc=GSE207652.].

Field-specific reporting

Please select the one below that is the best fit for your research. If you are not sure, read the appropriate sections before making your selection.

☒ Life sciences ☐ Behavioural & social sciences ☐ Ecological, evolutionary & environmental sciences

For a reference copy of the document with all sections, see nature.com/documents/nr-reporting-summary-flat.pdf

Life sciences study design

All studies must disclose on these points even when the disclosure is negative.

Sample size	No sample-size calculation was performed. Experiments compared a wild-type strains to mutants and were conducted on yeast cultures typically at the concentration of 1.10^7 cells/mL therefore examining at least 10^8 cells per condition. Based on previous publications (ex: PMID: 27863241),experiments were performed in triplicate or more as indicated in the manuscript, to enable statistical analysis.
Data exclusions	No data were excluded from analysis.
Replication	All experiments were repeated at least three times independently (biological replicates). qPCR were run as triplicates. All samples evaluated at the level of RNAseq were performed in triplicate. All information about number of experimental repeats are stated in legends.
Randomization	No deliberate randomization was performed. Standard methods were applied. For each mutant, independent positive transformants, randomly chosen, were examined in replicated experiments. For experiments involving plasmids, transformations were repeated and analyzed independently . Time-courses were replicated at different dates in different batch of media, thus guaranteeing minimal covariates.
Blinding	Not applicable. Experiments were not based on qualitative scoring metrics. Samples were collected and analyzed as "numbers" that were turned into strain identifiers and conditions at the end of the process.

Reporting for specific materials, systems and methods

We require information from authors about some types of materials, experimental systems and methods used in many studies. Here, indicate whether each material, system or method listed is relevant to your study. If you are not sure if a list item applies to your research, read the appropriate section before selecting a response.

Materials & experimental systems

n/a	Involved in the study	
☐	☒	Antibodies
☒	☐	Eukaryotic cell lines
☒	☐	Palaeontology and archaeology
☒	☐	Animals and other organisms
☒	☐	Human research participants
☒	☐	Clinical data
☒	☐	Dual use research of concern

Methods

n/a	Involved in the study	
☒	☐	ChIP-seq
☒	☐	Flow cytometry
☒	☐	MRI-based neuroimaging

Antibodies

Antibodies used	Peroxidase AffiniPure Goat Anti-Mouse IgG (H+L): Jackson ImmunoResearch - 115-035-003<br>Peroxidase AffiniPure Goat Anti-Rabbit IgG (H+L) Jackson ImmunoResearch - 115-035-144

Peroxidase AffiniPure Goat Anti-Rat IgG (H+L) Jackson ImmunoResearch - 115-035-143
Anti FLAG-M2 SIGMA-ALDRICH - F3165 Lot#SLBQ7119V
Anti-GFP: Takara - 632 381 Lot#A8034133
Anti-Kar2 HDEL: Santa Cruz - sc 53472 Lot# J1716
Anti-Pdi1: Invitrogen – MAI-10032 Lot#TD2558782
Anti-Pab1: Invitrogen – Abcam - ab 189635
Anti-Tub1: Santa Cruz - sc 53030 Lot#G1919
Anti-Hac1: a gift from Peter Walter = Rabbit anti-Hac1pi serum raised against a peptide corresponding to its last 10 C-terminal amino acids (CFELNDFFIT).
Anti RNA Polymerase II (8WG16) Eurogentec MMS-126P-050 Lot#PO18093

Validation

Antibodies have been validated by the manufacturer or by the provider:
- Anti-FLAG-M2 SIGMA-ALDRICH - F3165 validated for WB. https://www.sigmaaldrich.com/FR/fr/product/sigma/f3165
- Anti-GFP: Takara 632 381. Validated for WB. https://www.takarabio.com/search-results?term=632381&tab=product
- Anti-Kar2 HDEL: Santa Cruz - sc 53472. Validated for WB in yeast. https://www.scbt.com/p/hdel-antibody-2e7
- Anti-Pdi1: Invitrogen – MAI-10032 Validated for WB in yeast. https://www.thermofisher.com/antibody/product/PDI-Antibody-clone-38H8-Monoclonal/MA1-10032
- Anti-Pab1: Abcam - ab 189635 Validated for WB in yeast. PMID: 8413212.
- Anti-Tub1: Santa Cruz - sc 53030 Validated for WB in yeast https://www.scbt.com/fr/p/alpha-tubulin-antibody-yol1-34?requestFrom=search
-Anti RNA Polymerase II (8WG16) Eurogentec MMS-126P-050 Validated for ChIP in yeast. PMID: 11485988.
-Anti-Hac1 antibodies were previously validated PMID: 8898193.

- In addition, for tagged proteins, a "no tag" control was run to verify the tagging and the specificity of the antibody.
